# Microglia induce the transformation of A1/A2 reactive astrocytes via the CXCR7/PI3K/Akt pathway in chronic post-surgical pain

**DOI:** 10.1186/s12974-020-01891-5

**Published:** 2020-07-14

**Authors:** Ting Li, Tongtong Liu, Xuhui Chen, Li Li, Miaomiao Feng, Yue Zhang, Li Wan, Chuanhan Zhang, Wenlong Yao

**Affiliations:** 1 Department of Anesthesiology, Tongji Hospital, Tongji Medical College, Huazhong University of Science and Technology, Wuhan, 430030 Hubei Province People’s Republic of China; 2 Department of Ophthalmology, Tongji Hospital, Tongji Medical College, Huazhong University of Science and Technology, Wuhan, 430030 Hubei Province People’s Republic of China; 3 Department of Physiology, Hubei University of Chinese Medicine, Wuhan, 430065 Hubei Province People’s Republic of China

**Keywords:** Skin/muscle incision and retraction, A1 astrocytes, A2 astrocytes, Microglia, Chronic post-surgical pain

## Abstract

**Background:**

Activated astrocytes play important roles in chronic post-surgical pain (CPSP). Recent studies have shown reactive astrocytes are classified into A1 and A2 phenotypes, but their precise roles in CPSP remain unknown. In this study, we investigated the roles of spinal cord A1 and A2 astrocytes and related mechanisms in CPSP.

**Methods:**

We used a skin/muscle incision and retraction (SMIR) model to establish a rat CPSP model. Microglia, CXCR7, and the phosphoinositide 3-kinase/Akt (PI3K/Akt) signaling pathways were regulated by intrathecal injections of minocycline (a non-specific microglial inhibitor), AMD3100 (a CXCR7 agonist), and LY294002 (a specific PI3K inhibitor), respectively. Mechanical allodynia was detected with von Frey filaments. The changes in microglia, A1 astrocytes, A2 astrocytes, CXCR7, and PI3K/Akt signaling pathways were examined by enzyme-linked immunosorbent assay (ELISA), western blot, and immunofluorescence.

**Results:**

Microglia were found to be activated, with an increase in interleukin-1 alpha (IL-1α), tumor necrosis factor alpha (TNFα), and complement component 1q (C1q) in the spinal cord at an early stage after SMIR. On day 14 after SMIR, spinal cord astrocytes were also activated; these were mainly of the A1 phenotype and less of the A2 phenotype. Intrathecal injection of minocycline relieved SMIR-induced mechanical allodynia and reverted the ratio of A1/A2 reactive astrocytes. The expression of CXCR7 and PI3K/Akt signaling was decreased after SMIR, while they were increased after treatment with minocycline. Furthermore, intrathecal injection of AMD3100 also relieved SMIR-induced mechanical allodynia, reverted the ratio of A1/A2 reactive astrocytes, and activated the PI3K/Akt signaling pathway, similar to the effects produced by minocycline. However, intrathecal injection of AMD3100 did not increase the analgesic effect of minocycline. Last, LY294002 inhibited the analgesic effect and A1/A2 transformation induced by minocycline and AMD3100 after SMIR.

**Conclusion:**

Our results indicated that microglia induce the transformation of astrocytes to the A1 phenotype in the spinal cord via downregulation of the CXCR7/PI3K/Akt signaling pathway during CPSP. Reverting A1 reactive astrocytes to A2 may represent a new strategy for preventing CPSP.

## Introduction

Chronic postsurgical pain (CPSP) refers to pain in and around the surgical area that lasts longer than 2 months, excluding pain due to disease recurrence, inflammation, and other factors [1, 2]. About 10–50% of patients experience persistent pain after surgical procedures, such as thoracotomy, cesarean section, radical mastectomy, and inguinal hernia repair. CPSP seriously affects post-operative recovery and the quality of life of the patient [3–5]. However, there is no effective strategy for the treatment and prevention of CPSP. The mechanism underlying CPSP is complex and remains to be elucidated. Previous studies have shown that CPSP is a neuropathic pain caused mainly by surgical damage to peripheral nerves [6]. However, Flatters demonstrated that skin/muscle incision and retraction (SMIR)-induced CPSP lasted at least 22 days without nerve damage [3].

In recent years, much attention has been paid to the role of spinal glial cells in the development and maintenance of pain [7, 8]. Many studies have demonstrated the roles of activated astrocytes in various types of chronic pain, such as neuropathic pain [9, 10], inflammatory pain [11, 12], and bone cancer pain [13]. It has been reported that reactive astrocytes can be classified into A1 and A2 phenotypes, which provide neuroprotective and neurotoxic effects, respectively [14]. Recent studies have demonstrated that reactive astrocytes are involved in Parkinson’s disease, spinal cord injury [15, 16], and traumatic brain injury [17]. However, the roles of A1 and A2 astrocytes in CPSP are unclear.

It is known that activated microglia can induce the transformation of astrocytes into the A1 phenotype by releasing interleukin-1 alpha (IL-1α), tumor necrosis factor (TNF), and complement component 1q (C1q) [14]. Minocycline, a microglial inhibitor, could provide analgesic and anti-inflammatory effects in a variety of pain models, such as neuropathic pain models [18–20], inflammatory pain models [21], and bone cancer pain models [22]. However, it is yet to be determined whether the phenotypic transformation of A1 and A2 reactive astrocytes is mediated by microglia in CPSP.

CXCR7, a non-classical G-protein-coupled receptor, is involved in CXCL12-mediated cell cycle regulation and cell proliferation [23, 24]. AMD3100, a CXCR7 agonist, has been reported to induce the transformation of microglia into the anti-inflammatory M2 subtype and astrocytes into the neuroprotective A2 subtype [25, 26]. Studies have also documented that CXCR7 signaling plays a role in regulating cell proliferation and differentiation via the phosphoinositide 3-kinase/Akt (PI3K/Akt) signaling pathway [27, 28].

In this study, in order to identify the roles of different phenotypes of reactive astrocytes in CPSP, we first examined changes in A1 and A2 astrocytes in the spinal cord after SMIR. We then investigated the role of microglia in regulating A1/A2 transformation in CPSP, using minocycline. Last, we investigated the molecular mechanism by which microglia-mediated A1/A2 transformation occurred in CPSP by examining the CXCR7/PI3K/Akt signaling pathway.

## Material and methods

### Animals

Male Sprague-Dawley rats (200–220 g) were supplied from Tongji Medical College, Huazhong University of Science and Technology, Wuhan, Hubei Province, People’s Republic of China. The rats were housed under standard conditions (temperature: 22–25 °C, relative humidity: 45–65%, and 12-h light to dark cycle, with food and water ad libitum). All experiments were approved by the Experimental Animal Care and Use Committee of Tongji Medical College, Huazhong University of Science and Technology, and were in agreement with the National Institutes of Health Guidelines for the Care and Use of Laboratory Animals.

### Skin/muscle incision and retraction

The skin/muscle incision and retraction (SMIR) surgery was carried out, as previously reported [3]. Briefly, the medial skin of the thigh was cut 1.5–2 cm from approximately 4 mm medial to the saphenous vein to reveal the thigh muscles after rats were intraperitoneally injected with 1% sodium pentobarbital (50 mg/kg). The superficial layer of the gracilis muscle was cut 7–10 mm, and then, the muscle was further separated using a blunt dissection technique to allow the micro-dissecting retractor (Biomedical Research Instruments Inc., USA) to be inserted. All prongs of the micro-dissecting retractor were positioned below the superficial layer of the gracilis muscle; then, the skin and superficial muscles of the thigh were retracted by 2 cm to expose the underlying adductor fascia for 1 h. Isoflurane was used to provide additional anesthesia if necessary. The muscles and skin were sutured with 3.0 and 4.0 Vicryl sutures after 1 h. Large absorbent bench underpads were used to cover the rats to reduce the drying of the surgical site and heat loss from the rats. For the sham group, the skin and superficial muscles were cut without retraction.

### Intrathecal catheterization and drug administration

Intrathecal catheterization was carried out, as previously reported [22]. Briefly, the PE10 catheters (inner diameter 0.3 mm, outer diameter 0.6 mm) were inserted from L5–L6 spinous processes to the lumbar enlargement 5 days prior to the establishment of SMIR models. The rats were temporarily paralyzed after intrathecal injection of 2% lidocaine (10 μL), indicating the success of catheterization. All rats were allowed to recover for 5 days before experiments.

Minocycline hydrochloride (#M9511, Sigma-Aldrich, USA), a microglial inhibitor, was dissolved in saline and intrathecally injected at a dose of 100 μg/20 μL immediately and for seven consecutive days after SMIR. AMD3100 (S8030, Selleck Chemicals, USA), a CXCR7 agonist, was dissolved in saline and intrathecally injected at a dose of 20 μg/10 μL immediately and for seven consecutive days after SMIR. LY294002 (HY-10108, MCE, USA), a specific antagonist of PI3K, was dissolved in 10% dimethyl sulfoxide (DMSO) and injected intrathecally at a dose of 10 μg/5 μL 15 min before minocycline or AMD300 treatment. The doses of minocycline, AMD3100, and LY294002 were determined based on our preliminary studies and previous reports [22, 29, 30].

### Paw withdrawal threshold test

As described previously [3, 31], the mechanical paw withdrawal threshold (PWT) test was performed with von Frey filaments (Stoelting, Wood Dale, IL, USA) on days 0, 1, 4, 7, 14, and 21 at 09:00 AM. Briefly, the rats were placed in a separate transparent box with a wire mesh at the bottom that allowed the paws to be fully touched and then allowed to adapt for 40 min. Different von Frey filaments, ranging from 2 to 15 g, were gradually applied for up to 3 s to the mid-plantar area of the right hindpaw in an ascending manner. Sudden claw retraction, shaking, or licking was regarded as a positive reaction. The PWT was defined as the minimum force required to cause a positive reaction at least three times in five tests.

### Enzyme-linked immunosorbent assay analysis

The rats were sacrificed under deep anesthesia, and L3–L5 spinal segments were immediately removed and homogenized in phosphate-buffered saline (PBS). The supernatants of tissue homogenates were collected and analyzed using rat IL-1α (Elabscience Biotechnology Co., Ltd., Wuhan, China), TNF-α (Elabscience Biotechnology Co., Ltd.), or C1q (LifeSpan BioSciences Inc., USA) ELISA kits, according to the manufacturer’s instructions.

### Western blot analysis

The rats were sacrificed under deep anesthesia, and L3–L5 spinal segments were immediately removed and homogenized in an ice-cold mixture of radioimmunoprecipitation assay lysis buffer, phosphatase inhibitor, and phenylmethylsulfonyl fluoride (Boster Biological Technology, Wuhan, China) and then centrifuged at 12,000 rpm at 4 °C for 30 min. The protein concentration was determined using a BCA protein assay kit (Thermo Scientific, USA). The proteins were boiled at 90 °C in a loading buffer for 8 min and stored at − 80 °C until use. Samples (30–50 μg protein) were loaded and separated on 10% sodium dodecyl sulfate-polyacrylamide gel electrophoresis and then transferred to a polyvinylidene fluoride membrane. The membranes were blocked with 5% bovine serum albumin in Tris-buffered saline and Tween 20 (TBST, 0.1%) for 2 h at room temperature, followed by overnight incubation at 4 °C with specific primary antibodies: rabbit anti-C3/C3a antibody (A13283, 1:1000, Abclonal, Wuhan, China), rabbit anti-S100A10 antibody (ab187201, 1:500, Abcam, MA, USA), mouse anti-glial fibrillary acidic protein antibody (GFAP, #3670, 1:5000, Cell Signaling Technology, MA, USA), rabbit anti-CXCR7 antibody (ab72100, 1:1000, Abcam), rabbit anti-p-PI3K antibody (AF3241, 1:1000, Affinity, Wuhan, China), mouse anti-PI3K antibody (60225-1-Ig, 1:1000, Proteintech), mouse anti-p-Akt antibody (66444-1-Ig, 1:1000, Proteintech), rabbit anti-Akt antibody (10176-2-AP, 1:1000, Proteintech), and mouse anti-glyceraldehyde 3-phosphate dehydrogenase (GAPDH) antibody (AC002, 1:5000, Abclonal). After washing in TBST, the membranes were incubated with horseradish peroxidase (HRP)-conjugated goat anti-mouse secondary antibody (1:5000, Aspen, Wuhan, China) or goat anti-rabbit secondary antibody (1:5000, Aspen) for 2 h at room temperature. Finally, the protein bands were detected by SuperLumia ECL Plus HRP Substrate Kit (K22030, Abbkine, Wuhan, China) and exposed using ChemiDoc XRS+ imaging system (Bio-Rad, USA). The intensity of bands was analyzed using System with Image Lab software (Bio-rad Laboratories), standardized against GAPDH, and the band density of the sham group was set as 1.

### Immunofluorescence

The rats were transcardially perfused with PBS, followed by 4% neutral-buffered paraformaldehyde (PFA). Spinal cord tissue was removed from levels L3–L5, post-fixed overnight in 4% PFA, and then dehydrated in 30% sucrose solution for 2 days at 4 °C. The harvested spinal cord samples were sectioned to 20 μm thickness in a cryostat (CM1900, Leica, Germany). The sections were blocked with 5% donkey serum for 1 h at room temperature and incubated with goat anti-ionized calcium-binding adapter molecule 1 (Iba1, ab5076, 1:200, Abcam) overnight at 4 °C. Then, the sections were incubated with Alexa Fluor 488-labeled donkey anti-goat secondary antibody (1:100, Jackson ImmunoResearch, West Grove, PA, USA) for 2 h at room temperature.

For double immunofluorescence, the sections were blocked with 5% goat serum and 0.3% Triton X-100 in PBS for 1 h at room temperature and incubated with a mixture of rabbit anti-C3/C3a antibody (A13283, 1:100, Abclonal) and mouse anti-GFAP antibody (#3670, 1:200, Cell Signaling Technology)/goat anti-Iba1 antibody (ab5076, 1:200, Abcam)/mouse anti-neuronal nuclei antibody (NeuN, MAB377, 1:50, Millipore) or rabbit anti-S100A10 antibody (ab187201, 1:100, Abcam) and GFAP/Iba1/NeuN, or rabbit anti-CXCR7 antibody (ab72100; 1:50; Abcam) and GFAP/Iba1/NeuN. Then, the sections were incubated with a mixture of secondary antibodies, including Alexa Fluor 594-labeled goat anti-rabbit secondary antibody (1:400; Jackson ImmunoResearch) and Alexa Fluor 488-labeled goat anti-mouse secondary antibody (1:200; Jackson ImmunoResearch), for 2 h at room temperature. Images were captured using a fluorescence microscope (DM2500, Leica, Germany). As described previously [32, 33], the mean fluorescence intensity and the number of Iba1^+^ cells of microglia were calculated using Image J (National Institutes of Health, Bethesda, MD, USA).

### Experimental designs and animal groups

**Fig. 1:**
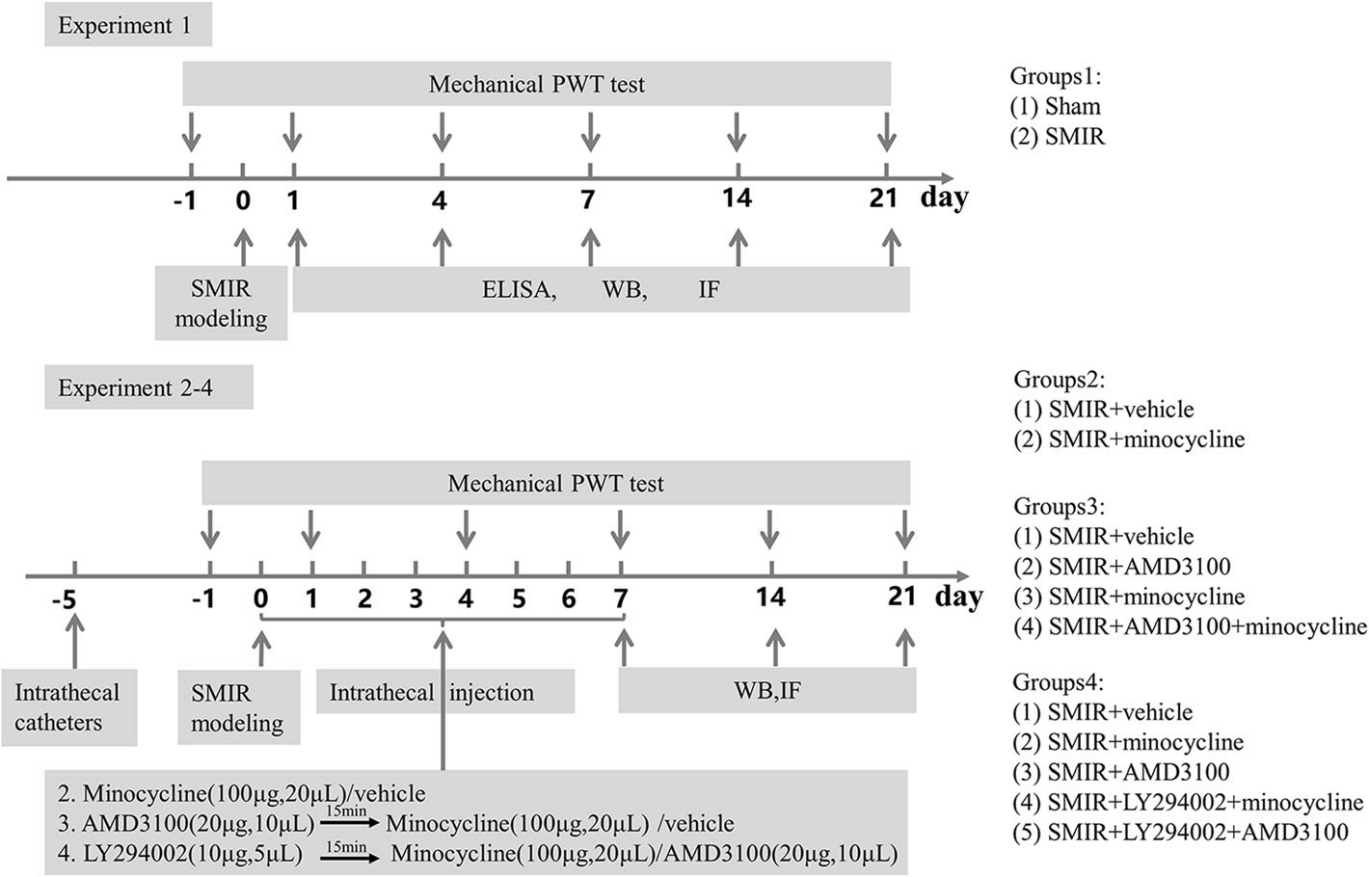
Experimental designs and animal groups. Experiment 1: changes in pain behavior and glial phenotypes after SMIR in rats. Experiment 2: effects of minocycline pretreatment on mechanical allodynia, glial phenotypes, and the CXCR7/PI3K/Akt pathway after SMIR in rats. Experiment 3: effects of CXCR7 agonist pretreatment on mechanical allodynia after SMIR in rats. Experiment 4: effects of PI3K/Akt pathway inhibitor pretreatment on the analgesic effect of minocycline and AMD3100. WB: western blot; IF: immunofluorescence; ELISA: enzyme-linked immunosorbent assay; SMIR: skin/muscle incision and retraction; AMD3100: CXCR7 agonist; minocycline: microglia inhibitor; LY294002: PI3K inhibitor; PWT: paw withdrawal threshold

As shown in Fig. 1, there were four experiments in this study.

#### Experiment 1: Changes in mechanical allodynia and glial expression after SMIR in rats

Sixty rats were randomly allocated to a sham or SMIR group. The PWTs were measured at days 1, 4, 7, 14, and 21 after surgery, and then, the L3–L5 region of the spinal cord was extracted for western blot and immunofluorescent analysis. According to previous studies and our preliminary studies, the activation of spinal microglia was most significant at day 7 after SMIR surgery, while the activation of spinal astrocytes in rats was most significant at day 14 after SMIR surgery [4, 34, 35]. Therefore, we chose the 7th day after the sham operation as a control to study microglial activation and the 14th day after the sham operation as a control to study astrocyte activation.

#### Experiment 2: The effects of minocycline pretreatment on mechanical allodynia, glial expression, and the CXCR7/PI3K/Akt pathway after SMIR

After SMIR surgery, 40 rats were divided into SMIR+vehicle and SMIR+minocycline groups. The PWTs were measured 30 min before each injection. The L3–L5 region of the spinal cord was collected from the SMIR+minocycline group at days 7, 14, and 21 after surgery, and SMIR+vehicle group at 14 days after surgery for western blot and immunofluorescence analysis.

#### Experiment 3: The effects of CXCR7 agonist pretreatment on mechanical allodynia after SMIR

After SMIR surgery, 24 rats were divided into four groups: SMIR+vehicle group, SMIR+minocycline group, SMIR+AMD3100 group, and SMIR+minocycline+AMD3100 group (*n* = 6 per group). The PWTs were measured 30 min before each injection. The L3–L5 region of the spinal cord was collected from each group 14 days after surgery for western blot and immunofluorescence analysis.

#### Experiment 4: The effects of PI3K/Akt pathway inhibitor pretreatment on the analgesic effect of minocycline and AMD3100

After SMIR surgery, 30 rats were randomly distributed into five groups: SMIR+vehicle group, SMIR+AMD3100 group, SMIR+minocycline group, SMIR+AMD3100+LY294002 group, and SMIR+minocycline+LY294002 group (*n* = 6 per group). The L3–L5 region of the spinal cord was collected 14 days after surgery for western blot and immunofluorescence analysis.

### Statistical analyses

All data are presented as the mean ± standard error of the mean (SEM). All statistical analyses were conducted using GraphPad Prism 6 (GraphPad Software, San Diego, CA, USA). Two-way repeated measures analysis of variance (ANOVA) followed by Bonferroni’s post hoc test was used for the analysis of the PWT data. One-way ANOVA, followed by Bonferroni’s post hoc test, was used for ELISA, immunofluorescence, and western blot data analysis. Differences with *P* < 0.05 were considered statistically significant.

## Results

### Microglia are activated in the spinal cord in the early stages of SMIR

**Fig. 2:**
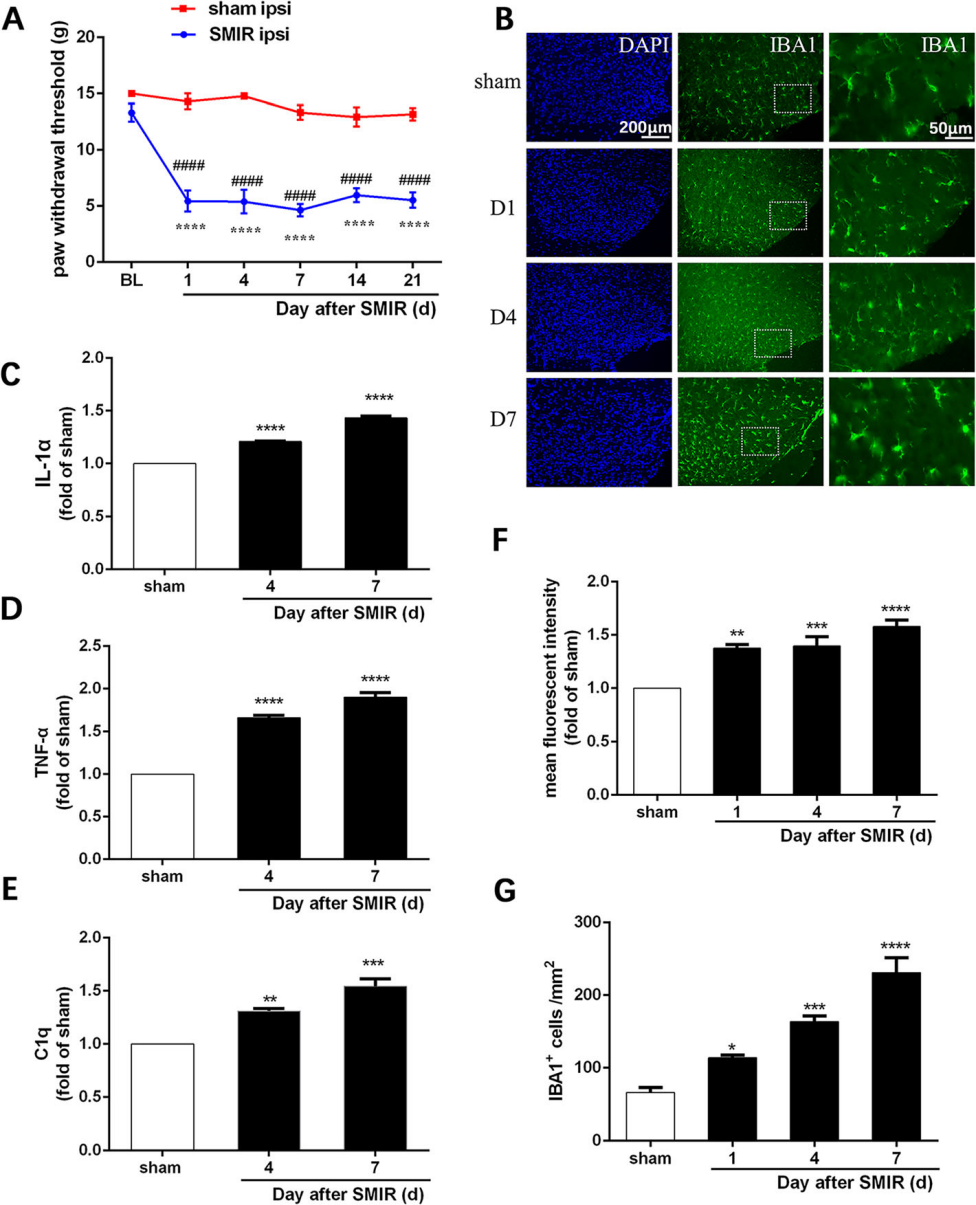
Microglia were activated in the spinal cord in the early stages of SMIR. **a**. Mechanical allodynia evaluated by the paw withdraw threshold (*n* = 10). **b** Representative images of immunofluorescence staining with Iba1 on lumbar spinal cord sections from rats (*n* = 4). Scale bar: 50 μm and 200 μm, respectively. **c–e** ELISA results for IL-1α, TNFα, and C1q in the spinal dorsal horn of rats (*n* = 3). The expressions of IL-1α, TNFα, and C1q in the sham group were set as 1 for quantification purposes. **f** Quantification of the mean fluorescent intensity of Iba1 positive cells in the spinal cord horn (*n* = 4). **g** Quantification of the number of Iba1 positive cells per square millimeter in the spinal cord horn (*n* = 4). Compared with sham rats, *P < 0.05, **P < 0.01, ***P < 0.001, ****P < 0.0001; compared with baseline, ^####^*P* < 0.0001. BL: baseline; d: day; DAPI: nuclear staining; IBA1: ionized calcium-binding adapter molecule 1; ipsi: ipsilateral; SMIR: skin/muscle incision and retraction

Behavioral testing showed that mechanical PWT was decreased in the ipsilateral hindpaw from days 1 to 21 in the SMIR group, compared with baseline. Compared to sham rats, the SMIR rats exhibited a decreased PWT in the ipsilateral hindpaw from days 1 to 21 (Fig. 2a, group: *F*(1, 9) = 136.3, *P* < 0.0001; time: *F*(5, 45) = 19.58, *P* < 0.0001; interaction: *F*(5, 45) = 10.47, *P* < 0.0001). These results indicated that the CPSP model was successfully induced by SMIR.

Microglial expression in the spinal dorsal horn was examined by immunofluorescent labeling of Iba1. As shown in Fig. 2b, microglia bodies were enlarged with retraction of the protuberances at days 1, 4, and 7 after SMIR. The results of the quantitative analysis showed that compared with the sham group, the mean fluorescent intensity and number of Iba1-positive cells in the spinal cord were significantly increased after SMIR (Fig. 2f, *F*(3, 12) = 19.1, *P* < 0.0001; Fig. 2g, *F*(3, 12) = 35.83, *P* < 0.0001). The results of the ELISA showed that spinal cord IL-1α, TNFα, and C1q were significantly higher in the SMIR group than in the sham group (Fig. 2c–e, IL-1α: *F*(2, 6) = 510, *P* < 0.0001; TNFα: *F*(2, 6) = 536.2, *P* < 0.0001; C1q: *F*(2, 6) = 41.76, *P* = 0.0003). These indicated that microglia were activated in the spinal cord in the early stages of SMIR.

### Astrocytes were activated and mainly expressed as the A1 phenotype in the spinal cord after SMIR

**Fig. 3.**
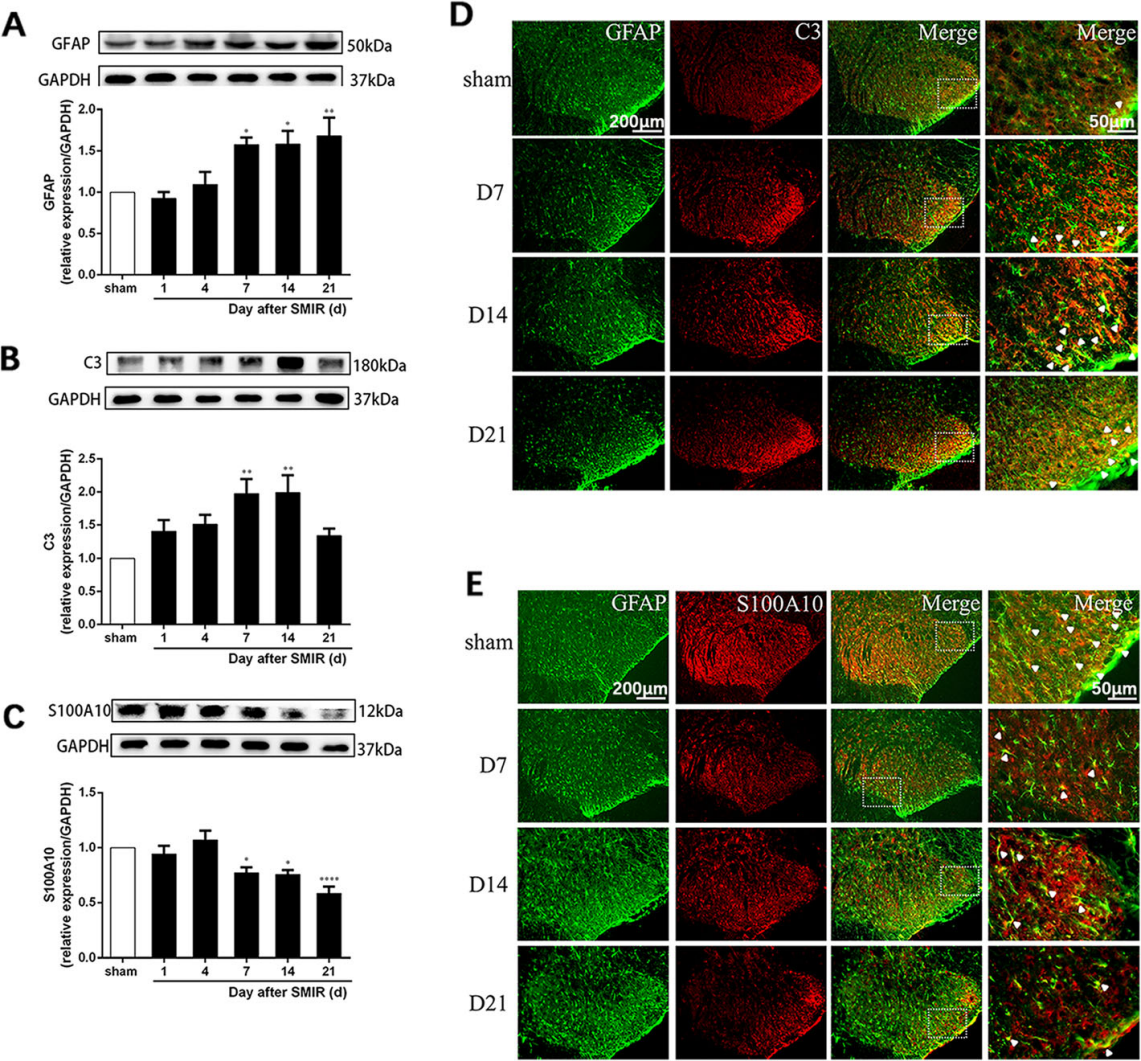
Astrocytes were activated and mainly expressed as the A1 phenotype in the spinal cord after SMIR. **a**–**c** Representative blots and quantification of GFAP, C3, and S100A10 in the spinal cord of sham (day 14) and SMIR group rats (*n* = 7). The expressions of GFAP, C3, and S100A10 were normalized to GAPDH for each sample. GFAP, C3, and S100A10 levels in the sham group were set as 1 for quantification. Compared with the sham rats, *P < 0.05, **P < 0.01, ****P < 0.0001. **d**–**e** Double immunofluorescence staining for C3 (red) or S100A10 (red) and GFAP (green) in the spinal cords of sham (day 14) and SMIR group rats (*n* = 3). Scale bar: 50 μm or 200 μm. d: day; GFAP: glial fibrillary acidic protein; SMIR: skin/muscle incision and retraction

The changes in astrocytes, A1 versus A2, after SMIR were examined by western blot with GFAP, C3, and S100A10. The results show that compared with the sham group, GFAP increased in a time-dependent manner at days 7, 14, and 21 (Fig. 3a, *F*(5, 36) = 6.295, *P* = 0.0003). The expression of C3 (a marker for the A1 astrocyte phenotype) was significantly increased at days 7 and 14 after SMIR (Fig. 3b, *F*(5, 36) = 5.004, *P* = 0.0014). In contrast, the expression of S100A10 (a marker for the A2 astrocyte phenotype) was decreased at days 7, 14, and 21 after SMIR (Fig. 3c, *F*(5, 36) = 9.672, *P* < 0.0001).

We further examined the expression of A1 and A2 phenotypes in the spinal cord by double immunofluorescence. As shown in Fig. 3d, e, C3 and S100A10 were mostly colocalized with GFAP in the superficial region of the spinal cord of the SMIR rats. Compared with sham rats, A1 reactive astrocytes were increased, while A2 reactive astrocytes were decreased 14 days after SMIR. This indicated that reactive astrocytes were mainly expressed as the A1 phenotype in the spinal cord after SMIR.

### Minocycline reverted the A1/A2 ratio of reactive astrocytes and relieved mechanical allodynia after SMIR in rats

**Fig. 4:**
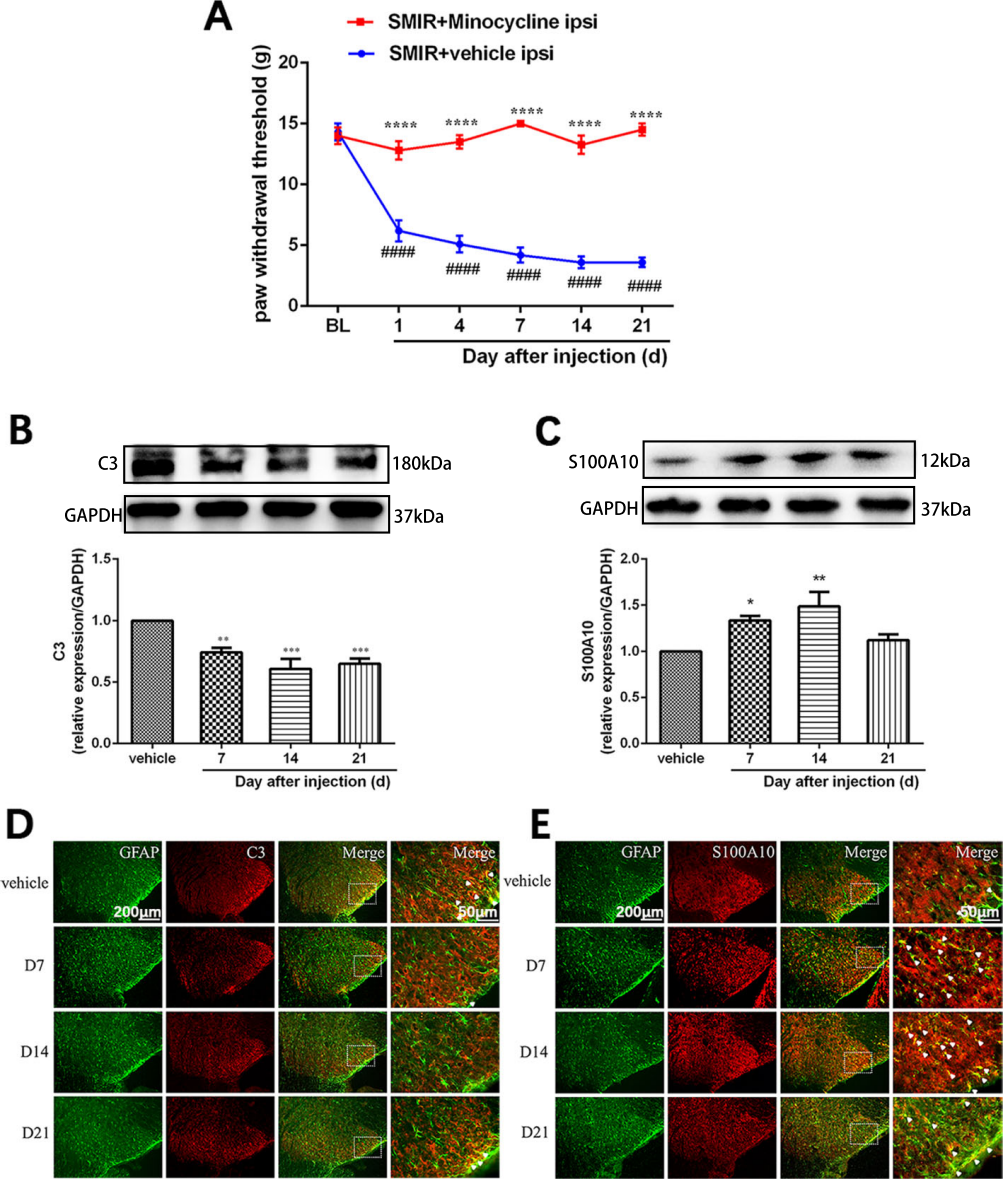
Minocycline reverted the A1/A2 ratio of reactive astrocytes and relieved mechanical allodynia in rats after SMIR. Minocycline or vehicle was intrathecally injected to rats immediately and for seven consecutive days after SMIR. **a** Mechanical allodynia was evaluated using paw withdraw threshold tests at days 1, 4, 7, 14, and 21 after injection with vehicle or minocycline (*n* = 10). **b**, **c** Representative blots and quantification of C3 and S100A10 in the spinal cords of SMIR rats after injection with vehicle or minocycline (*n* = 5). Compared with the SMIR+vehicle group, *P < 0.05, **P < 0.01, ***P < 0.001, ****P < 0.0001; compared with baseline, ^####^*P* < 0.0001. **d**, **e** Double immunofluorescence staining for C3 (red) or S100A10 (red) and GFAP (green) in the spinal cords of the SMIR+vehicle group rats at day 14 after surgery or SMIR+minocycline rats at days 7, 14, and 21 after surgery (*n* = 3). BL: baseline; d: day; GFAP: glial fibrillary acidic protein; ipsi: ipsilateral; SMIR: skin/muscle incision and retraction

Minocycline was intrathecally injected immediately and for seven consecutive days after SMIR to investigate the role of microglia in regulating the A1/A2 phenotypic transformation in CPSP. As shown in Fig. 4a, behavioral testing showed that PWT was significantly increased after intrathecal injection of minocycline, compared with the SMIR+vehicle group (group: *F*(1, 9) = 223.2, *P* < 0.0001; time: *F*(5, 45) = 21.52, *P* < 0.0001; interaction: *F*(5, 45) = 33.11, *P* < 0.0001).

Western blot analysis showed that the expression of C3 (a marker for the A1 astrocyte phenotype) was significantly downregulated on days 7, 14, and 21 after minocycline treatment (Fig. 4b, *F*(3, 16) = 12.64, *P* = 0.0002), while the expression of S100A10 (a marker for the A2 astrocyte phenotype) was upregulated on days 7 and 14 after minocycline treatment (Fig. 4c, *F*(3, 16) = 6.082, *P* = 0.0058). Similarly, double immunofluorescence showed that compared with the SMIR+vehicle group, the expression of the A1 astrocytic phenotype was significantly decreased, while the expression of the A2 astrocytic phenotype was significantly increased in the SMIR+minocycline group (white arrow in Fig. 4d, e). These results demonstrated that inhibition of microglial activation reverted the A1/A2 ratio of reactive astrocytes and relieved mechanical allodynia after SMIR in rats.

### The CXCR7 and PI3K/Akt signaling pathways are involved in CPSP

**Fig. 5:**
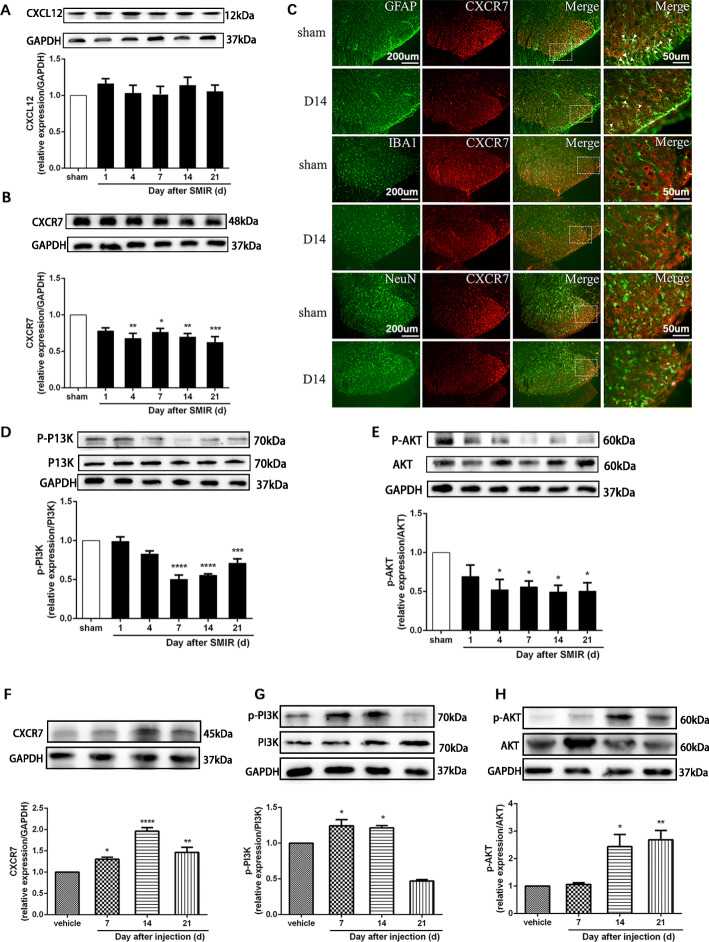
The CXCR7 and PI3K/Akt signaling pathways are involved in CPSP in the dorsal horn of the spinal cord. **a**, **b**, **d**, **e** Representative western blot images and quantification of CXCL12 (**a**), CXCR7 (**b**), p-PI3K (**d**), and p-Akt (**e**) in the spinal cords of sham and SMIR group rats (*n* = 4). **c** Double immunofluorescence staining for CXCR7 (red) labeling with GFAP (green) for astrocytes, Iba1 (green) for microglia, or NeuN (green) for neurons in sham and SMIR rats at day 14 after surgery (*n* = 3). Scale bar: 50 μm or 200 μm. **f**–**h** Representative western blot images and quantification of CXCR7, p-PI3K, and p-Akt in the spinal cords of SMIR rats after injection of vehicle or minocycline (*n* = 3–4). Compared with the sham rats, *P < 0.05, **P < 0.01, ***P < 0.001, ****P < 0.0001. The expression of CXCR7 was normalized to GAPDH for each sample, and p-P13K and p-Akt were normalized to PI3K and Akt, respectively, for each sample. The fold change of CXCR7, p-PI3K, and p-Akt in the sham group was set as 1 for quantification. d: day; GFAP: glial fibrillary acidic protein; IBA1: ionized calcium-binding adapter molecule 1; NeuN: neuronal nuclei; SMIR: skin/muscle incision and retraction

The molecular mechanisms of microglia-regulated astrocyte polarization in CPSP remained unclear in the studies above. CXCR7 is involved in inflammatory communication between glial cells and neurons, which regulates the polarization of glial cells. Therefore, we first examined changes in CXCR7 and its endogenous ligand CXCL12 in the spinal cords of SMIR rats. Double immunofluorescence staining showed that CXCR7 was expressed mostly in astrocytes and, to a small extent, in microglia and neurons (Fig. 5c). As shown in Fig. 5a, b, western blot analysis showed that compared with the sham group, the expression of CXCR7 was reduced in the spinal cord in the SMIR group (*F*(5, 18) = 5.831, *P* = 0.0023), while the expression of its ligand CXCL12 was unchanged. In contrast, after treatment with minocycline, the expression of CXCR7 was significantly increased at days 7, 14, and 21 (Fig. 5f, *F*(3, 12) = 28.04, *P* < 0.0001).

The PI3K/Akt pathway was examined as it is an important signaling pathway that regulates cell growth, differentiation, and migration. Western blot analysis showed that compared with the sham group, the expression of p-PI3K was significantly downregulated at days 7, 14, and 21 after SMIR (Fig. 5d, *F*(5, 18) = 24.02, *P* < 0.0001), which was reversed after minocycline treatment (Fig. 5g, *F*(3, 12) = 57.38, *P* < 0.0001). The expression of p-Akt was significantly downregulated at days 4, 7, 14, and 21 after SMIR (Fig. 5e, *F*(5, 18) = 3.558, *P* = 0.0206), which was reversed after minocycline treatment (Fig. 5h, *F*(3, 8) = 10.16, *P* = 0.0042). These results indicate that minocycline can prevent SMIR-induced downregulation of the CXCR7 and PI3K/Akt signaling pathways.

### The CXCR7 agonist AMD3100 has similar effects to minocycline in SMIR but has no synergistic effects when combined with minocycline

**Fig. 6:**
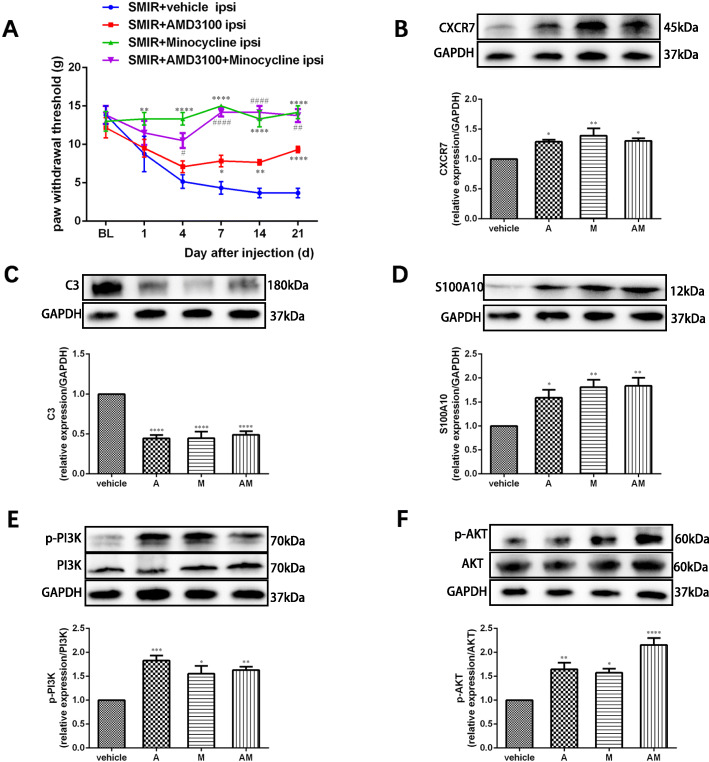
A CXCR7 agonist provided similar effects to minocycline after SMIR, but there was no synergistic effect with minocycline. AMD3100 alone or combined with minocycline was intrathecally injected immediately and for seven consecutive days after SMIR. **a** Mechanical allodynia was evaluated by paw withdraw threshold tests (*n* = 6). **b**–**f** Representative western blot images and quantification of CXCR7, C3, S100A10, p-PI3K, and p-Akt in the spinal cords of animals from different groups (*n* = 4–5). Compared with the SMIR+vehicle group, *P < 0.05, **P < 0.01, ***P < 0.001, ****P < 0.0001; compared with the SMIR+AMD3100+minocycline group, ^#^*P* < 0.05, ^##^*P* < 0.01, ^####^*P* < 0.0001. A: AMD3100, a specific agonist of CXCR7; M: minocycline, an inhibitor of microglia; AM: AMD3100+minocycline; BL: baseline; d: day; ipsi: ipsilateral; SMIR: skin/muscle incision and retraction

To further investigate the role of CXCR7 in the microglia-mediated phenotypic transformation of reactive astrocytes, the specific CXCR7 agonist AMD3100 was intrathecally injected alone or in combination with minocycline immediately and for seven consecutive days after SMIR. As shown in Fig. 6a, behavioral testing showed that the PWT was significantly increased after intrathecal injections of AMD3100. However, the analgesic effect of AMD3100 alone was not comparable with that of minocycline alone or in combination with AMD3100 (group: *F*(3, 15) = 27.32, *P* < 0.0001; time: *F*(5, 25) = 7.841, *P* = 0.0001; interaction: *F*(15, 75) = 6.502, *P* < 0.0001). Western blot analysis showed that after injection of AMD3100, minocycline, or both, the expressions of CXCR7, p-PI3K, and p-Akt were significantly increased in the spinal cords of SMIR rats (Fig. 6b, for CXCR7: *F*(3, 16) = 6.523, *P* = 0.0043; Fig. 6e, for p-PI3K: *F*(3, 12) = 12.07, *P* = 0.0006; Fig. 6f, for p-Akt: *F*(3, 12) = 19.06, *P* < 0.0001). Moreover, the expression of C3 (a marker for the A1 astrocyte phenotype) was significantly downregulated (Fig. 6c, *F*(3, 16) = 28.07, *P* < 0.0001), while the expression of S100A10 (a marker for the A2 astrocyte phenotype) was upregulated (Fig. 6d, *F*(3, 16) = 9.484, *P* = 0.0008).

These results suggest that CXCR7 is one of the important downstream receptors for minocycline-induced behavioral improvement, upregulation of the PI3K/Akt signaling pathway, and transformation of astrocytes into the A2 subtype.

### LY294002, a specific PI3K inhibitor, inhibited the analgesic effect and A1/A2 transformation induced by minocycline and AMD3100 after SMIR

**Fig. 7:**
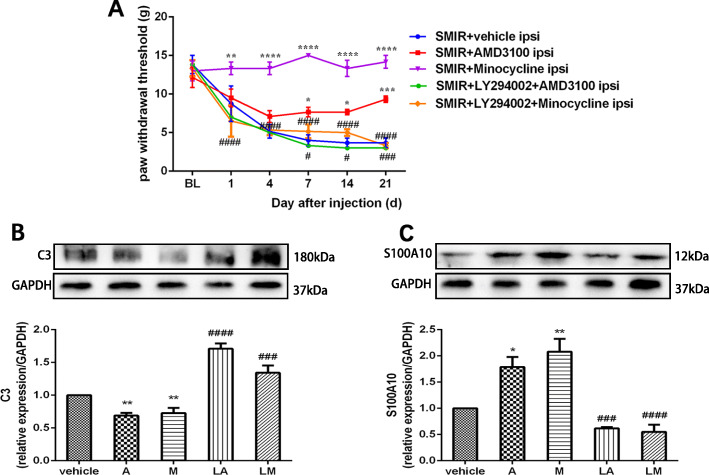
Minocycline and AMD3100 reverted the A1/A2 ratio of reactive astrocytes and improved behavior via PI3K/Akt pathway activation after SMIR. AMD3100 or minocycline was intrathecally injected immediately and for seven consecutive days after SMIR. Rats were administered intrathecal LY294002 before minocycline or AMD3100 administration. **a** Mechanical allodynia was evaluated by paw withdraw threshold tests (*n* = 6). **b**, **c** Representative western blot images and quantification of C3 and S100A10 in the spinal cords of animals from different groups (*n* = 5). Compared with the SMIR+vehicle group, *P < 0.05, **P < 0.01, ***P < 0.001, ****P < 0.0001; compared with the SMIR+AMD3100 group or the SMIR+minocycline group, ^#^*P* < 0.05, ^###^*P* < 0.001, ^####^*P* < 0.0001. A: AMD3100, a specific agonist of CXCR7; M: minocycline, an inhibitor of microglia; L: LY294002, a specific inhibitor of PI3K; LA: LY294002+AMD3100; LM: LY294002+minocycline; BL: baseline; d: day; ipsi: ipsilateral; SMIR: skin/muscle incision and retraction

To verify whether the PI3K/Akt pathway is the downstream mechanism by which microglia and CXCR7 regulate the transformation of reactive astrocytes during CPSP, LY294002, a specific PI3K inhibitor, was intrathecally administered prior to minocycline or AMD3100 injection. As shown in Fig. 7a, behavioral tests showed that the administration of LY294002 significantly antagonized the analgesic effects of minocycline or AMD3100 (group: *F*(1, 5) = 71.17, *P* = 0.0004; time: *F*(5, 25) = 5.182, *P* = 0.0021; interaction: *F*(5, 25) = 11.37, *P* < 0.0001, vs SMIR+minocycline group; group: *F*(1, 5) = 28.89, *P* = 0.003; time: *F*(5, 25) = 15.41, *P* < 0.0001; interaction: *F*(5, 25) = 3.912, *P* = 0.0093, vs SMIR+AMD3100 group). Moreover, the western blot results showed that LY294002 pre-treatment significantly hindered the downregulation of C3 (a marker for the A1 astrocyte phenotype) (Fig. 7b, *F*(4, 20) = 35.28, *P* < 0.0001) and the upregulation of S100A10 (a marker for the A2 astrocyte phenotype) (Fig. 7c, *F*(4, 20) = 20.93, *P* < 0.0001) induced by minocycline or AMD3100. These results suggested that minocycline and AMD3100 reverted the A1/A2 ratio of reactive astrocytes and relieved the mechanical allodynia induced by SMIR via the PI3K/Akt pathway.

## Discussion

In the present study, we demonstrated that (1) during SMIR-induced CPSP, astrocytes were activated and mainly manifested as the A1 phenotype after the activation of microglia in the spinal cord; (2) minocycline, a microglial inhibitor, relieved SMIR-induced mechanical allodynia and reverted the ratio of A1/A2 reactive astrocytes; (3) AMD3100, a CXCR7 agonist, relieved SMIR-induced mechanical allodynia, reverted the ratio of A1/A2 reactive astrocytes, and activated the PI3K/Akt signaling pathway, which was similar to the effects produced by minocycline; however, intrathecal injection of AMD3100 did not increase the analgesic effect of minocycline; and (4) LY294002, a specific PI3K inhibitor, inhibited the analgesic effect and A1/A2 transformation induced by minocycline and AMD3100 after SMIR. These results indicate that A1 and A2 astrocytes play different roles in the development of CPSP. The transformation of A1/A2 astrocytes during the development of CPSP was regulated by microglia, and the CXCR7/PI3K/Akt pathway is considered to be the downstream mechanism involved.

Previous studies have shown that SMIR induces CPSP that lasts at least 22 days without nerve injury [3]. Three rat pain models are similar to the SMIR model: the thoracotomy model, the gastrocnemius incision model, and the paw incision model. Unlike the thoracotomy model, where nerve degeneration leads to persistent pain [36], the SMIR model causes persistent pain without nerve injury. In addition, the paw incision model and gastrocnemius incision model induce mechanical allodynia for up to 4 days and 8 days, respectively, while the SMIR model induces mechanical allodynia for at least 3 weeks [3]. Therefore, we used the SMIR model in this study and found that it could induce persistent post-operative pain.

It is known that activated astrocytes are involved in the maintenance of chronic pain [7, 37]. Recent studies have also demonstrated that reactive astrocytes have two different phenotypes, A1 and A2, which play neurotoxic and neuroprotective roles, respectively [14]. Neurotoxic A1 astrocytes, which secrete toxic factors that kill mature oligodendrocytes and neurons, have been shown to be involved in a variety of neurodegenerative diseases [38] and spinal cord injury [39]. The detrimental role of A1 astrocytes in traumatic brain injury has also been well documented [40]. A2 reactive astrocytes have been shown to play a neuroprotective role in traumatic brain injury [17]. In the present study, we observed an imbalanced astrocytic polarization of A1 and A2 in the spinal cord of the rat SMIR model. During the development of CPSP, reactive astrocytes were mainly expressed as A1 astrocytes, with very few A2 astrocytes found. When mechanical allodynia was relieved in SMIR rats by intrathecal injection of minocycline or AMD3100, the expression of A1 astrocytes was decreased, and the expression of A2 astrocytes was increased. These results indicate that A1 astrocytes contribute to pain development, while A2 astrocytes are beneficial for relieving pain. Regulating the ratio of A1/A2 astrocytes may represent a new strategy for preventing CPSP.

A previous study showed that A1 astrocytes were induced by activated microglia via the secretion of IL-1α, TNFα, and C1q [14]. Qian et al. found that microglia induced the formation of A1 astrocytes by activating the Notch-Stat3 pathway in spinal cord injury [16]. Microglia induced the transformation of astrocytes into a neuroprotective phenotype by downregulating the P2Y1 receptor after brain trauma [41]. A2 astrocytes are induced by damaged neurons via the secretion of prokineticin-2 [42]. Milk fat globule epidermal growth factor 8 can decrease the expression of A1 astrocytes and increase the expression of A2 astrocytes by upregulating the activation of the PI3K/Akt pathway and downregulating the activation of the nuclear factor kappa-light-chain-enhancer of activated B cell pathway [43]. In addition, many studies suggested that microglia were activated in the early stages of chronic pain and thus appear to be involved in the initiation of chronic pain [44–47]. Consistent with this evidence, we found that microglial activation occurred earlier than astrocytic activation after SMIR and was accompanied by increased IL-1α, TNFα, and C1q levels in the spinal cord. Moreover, reactive astrocytes were mainly expressed as the A1 phenotype in the spinal cord after SMIR. These results suggested that A1 reactive astrocytes were induced by microglia during CPSP.

Minocycline, which selectively inhibits the activation of microglia, has no direct effect on neurons or astrocytes [44]. As a second-generation tetracycline, it has antibacterial and anti-inflammatory effects and also has antioxidant and anti-apoptotic effects [48]. In addition, minocycline is emerging as a promising therapy for chronic pain due to its good lipid solubility, easy passage through the blood-brain barrier, and analgesic effects in a variety of pain models [44, 49]. Choi et al. have shown that minocycline alleviated the development of mirror pain by inhibiting the production of IL-1β from microglia and suppressing astrocytic activation in inflammatory pain models [21]. In line with this, our results show that the intrathecal injection of minocycline prevents SMIR-induced mechanical allodynia. A1 astrocytes were significantly downregulated, and A2 astrocytes were dramatically upregulated after intrathecal injections of minocycline. These results indicated that minocycline relieved CPSP by reverting the ratio of A1/A2 astrocytes. They also confirmed that A1 reactive astrocytes were induced by microglia during CPSP.

CXCR7 is involved in inflammatory communication between glial cells and neurons, which regulates the polarization of glial cells. Odemis et al. found that CXCR7 was expressed on the surface of astrocytes in the CNS [50]. However, some studies found that CXCR7 was expressed in both astrocytes and microglia [51]. CXCR7 promotes cell maturation, differentiation, polarization, and migration when combined with its ligand CXCL12 [52, 53]. In addition, studies have shown that G-protein-coupled receptor kinase 2 in microglia regulates the signaling of CXCL12-bound CXCR7 by silencing and internalizing CXCR7 in astrocytes [54]. Evidence indicates that activation of CXCR7 can generate neuroprotective effects in the CNS by inhibiting the activation of microglia and astrocytes as well as by modulating M1/M2 microglial polarization and A1/A2 astrocyte transformation [25]. Our results showed that CXCR7 was predominantly colocalized with GFAP, with some colocalized with Iba1 or NeuN. The expression of CXCR7 decreased after SMIR, while it increased after minocycline pretreatment in SMIR rats, indicating that minocycline could regulate the expression of CXCR7 during CPSP.

Next, the CXCR7 agonist AMD3100 was injected intrathecally into SMIR rats, either alone or in combination with minocycline. Our results showed that AMD3100 attenuated SMIR-induced mechanical allodynia. CXCR7 was upregulated, A1 astrocytes were increased, and A2 astrocytes were decreased after intrathecal injection of AMD3100. However, we found that the analgesic effect of AMD3100 alone was less than that of minocycline alone or in combination with AMD3100. This suggests that there may be other mechanisms involved in the analgesic effect of minocycline, apart from CXCR7. For example, in sciatic nerve ligation, minocycline alleviates neuropathic pain by upregulating glutamate transporters in glial cells and preserving the normal activation of NMDA receptors in sensory synapses in the spinal cord [20].

Numerous studies have documented that CXCR7 signaling plays a role in promoting cell proliferation and differentiation through the PI3K/Akt signaling pathway [27, 28]. The PI3K/Akt signaling pathway is an important downstream pathway of mGluR5-mediated neuroprotection after cerebral ischemia [55]. Similarly, the PI3K/Akt pathway is essential for minocycline to reduce ketamine-induced neurological damage by promoting neural stem cell differentiation to neurons and inhibiting ketamine-induced cell apoptosis [56]. In this study, we found that the PI3K/Akt pathway was significantly downregulated during the development of CPSP, which was reversed after minocycline or AMD3100 treatment. LY294002, a specific PI3K inhibitor, antagonized the analgesic effect of minocycline and AMD3100 after SMIR. These results suggested that minocycline and AMD3100 relieved mechanical allodynia after SMIR via the PI3K/Akt pathway.

In an Alzheimer’s disease model, the transformation of reactive astrocytes to the A1 phenotype was accompanied by reduced activation of the PI3K/Akt pathway, while transformation to the A2 phenotype was accompanied by increased activation of the PI3K/Akt pathway [43, 57]. In line with this, we found that during the development of CPSP, an increase in A1 astrocytes was accompanied by reduced activation of the PI3K/Akt pathway, while the decrease in A1 astrocytes induced by AMD3100 and minocycline was accompanied by increased activation of the PI3K/Akt pathway. In addition, LY294002 pretreatment eliminated the decrease in the number of A1 astrocytes and the increase in the number of A2 astrocytes induced by minocycline and AMD3100 in the spinal cord of SMIR rats. Therefore, these results indicate that the PI3K/Akt signaling pathway is an essential downstream mechanism by which microglia and CXCR7 regulate the transformation of reactive astrocytes during CPSP.

There is a limitation to our study. Due to the lack of effective CXCR7 inhibitors, we did not investigate the effect of inhibition of CXCR7 signaling on the analgesic effect of minocycline and A1/A2 expression. Although CXCR7 small interfering RNA or CXCR7 knockout animals can be considered to further confirm the function of CXCR7 as the downstream mediator in microglia, our results suggest that there may be other mechanisms involved in the induction of A1 astrocytes by microglia. For example, Zou et al. demonstrated that the inhibition of the fibroblast growth factor 2/fibroblast growth factor 2, receptor 1 (FGF2/FGFR1) pathway resulted in an increase in A1 astrocytes after infrasound damage [58]. Therefore, there are other receptors and signaling pathways related to the unbalanced polarization of astrocytes toward the A1 phenotype in the development of CPSP, which requires further study.

This study demonstrated the different roles of A1 and A2 astrocytes in the development of CPSP, but the detailed mechanism by which they regulate pain was not explored. A previous study showed that mitochondrial dysfunction in A1 astrocytes caused inflammation and neuronal death, which promoted the development of neurodegenerative diseases [59]. In rat models of Alzheimer’s disease, A1 reactive astrocytes may drive neuronal death by releasing D-serine, which is the adjunctive agonist for excess glutamate to activate extrasynaptic *N*-methyl-d-aspartate receptors [60]. Bone mesenchymal stem cell-derived exosomes have been shown to inhibit the production of A1 neurotoxic astrocytes, accompanied by decreased neuronal apoptosis, axonal regeneration, and functional recovery after spinal cord injury [61]. Therefore, A1 astrocytes may promote the development of CPSP by releasing D-serine and pro-inflammatory factors that impair synaptic plasticity as well as by inducing neuronal and axonal injury and neuroinflammation. In contrast, A2 astrocytes promote neuronal survival and tissue repair by secreting neuroprotective factors. Studies have shown that the increased reactivity of A2 astrocytes was accompanied by improvements in mitochondrial energy metabolism, a decrease in pro-inflammatory factors, and an increase in neuroprotective factors such as brain-derived neurotrophic factor and glial cell-derived neurotrophic factor [42, 59, 62]. Therefore, the role of A1 astrocytes in regulating synaptic plasticity, energy metabolism, and neuroinflammation during chronic pain and the mechanism of A2 astrocytes in inhibiting the development of pain require further study.

## Conclusion

**Fig. 8:**
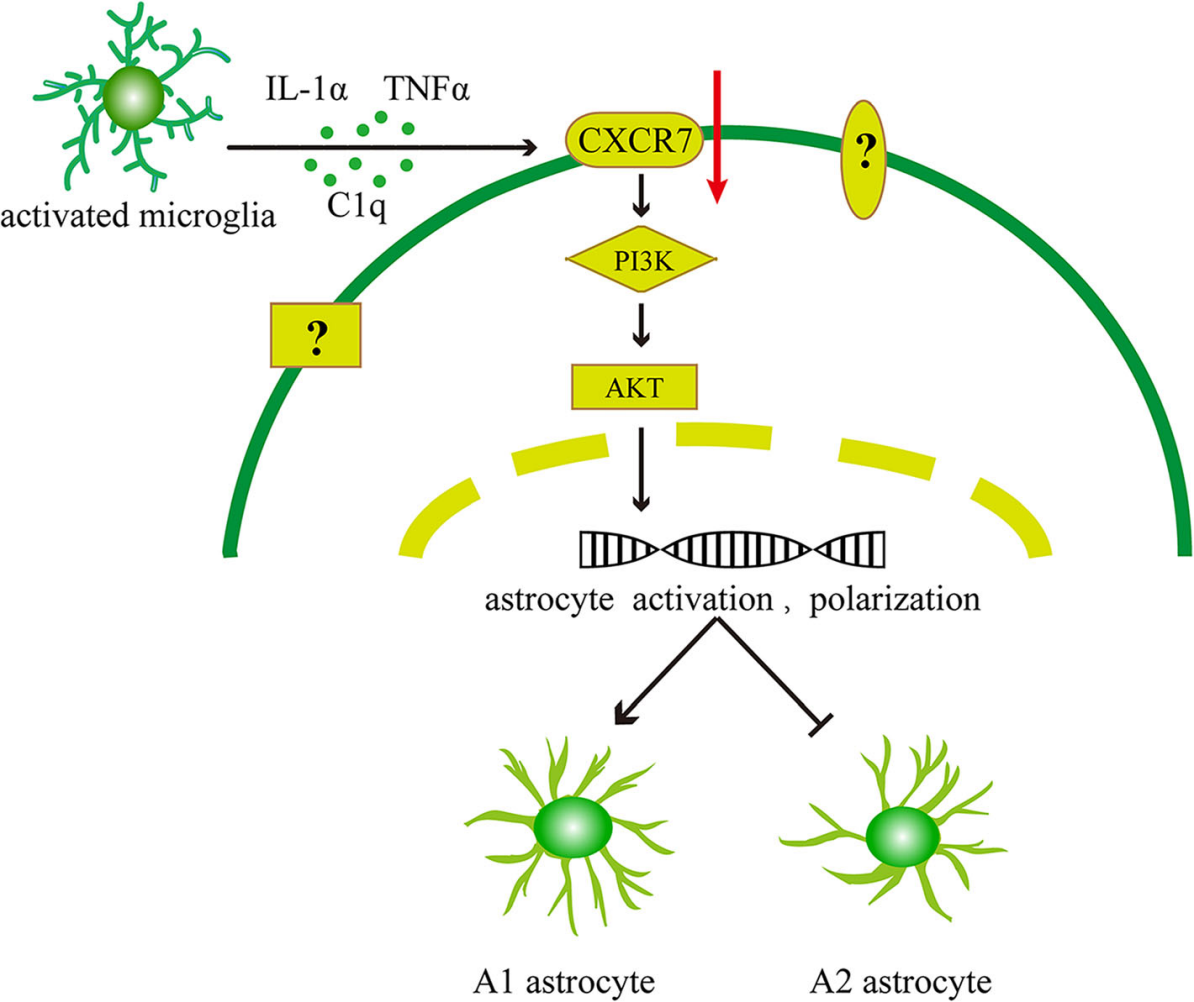
Schematic diagram demonstrating that microglia induce the transformation of A1/A2 reactive astrocytes via the CXCR7/PI3K/Akt pathway. During the development of CPSP, microglia are activated first and secrete cytokines such as IL-1α, TNF-α, and C1q, which leads to the downregulation of the CXCR7 receptor and the PI3K/Akt signaling pathway and activate astrocytes, inducing an increase in A1 astrocytes and a decrease in A2 astrocytes. There are other receptors and signaling pathways related to the imbalance in the polarization of astrocytes toward the A1 phenotype during chronic pain, which requires further study

This study is the first to show that A1 astrocytes contribute to the development of CPSP, while A2 astrocytes are beneficial for relieving CPSP. Microglia induced the transformation of astrocytes to the A1 phenotype in the spinal cord by reducing the activation of the CXCR7/PI3K/Akt signaling pathway during CPSP (Fig. 8). Reverting A1 reactive astrocytes to A2 astrocytes may represent a new strategy for preventing CPSP. However, the precise roles of A1 and A2 astrocytes in regulating CPSP require further study.

## Data Availability

The data and materials supporting the conclusions of this study are available from the corresponding author on reasonable request.

## Abbreviations

A: AMD3100
ANOVA: Analysis of variance
BL: Baseline
C1q: Complement component 1q
CPSP: Chronic post-surgical pain
DAPI: Nuclear staining
DMSO: Dimethyl sulfoxide
ELISA: Enzyme-linked immunosorbent assay
FGF2: Fibroblast growth factor 2
FGFR1: Fibroblast growth factor 2, receptor 1
GAPDH: Glyceraldehyde 3-phosphate dehydrogenase
GFAP: Glial fibrillary acidic protein
HRP: Horseradish peroxidase
Iba1: Ionized calcium-binding adapter molecule 1
IF: Immunofluorescence
IL-1α: Interleukin-1 alpha
ipsi: Ipsilateral
LA: LY294002 + AMD3100
LM: LY294002 + minocycline
M: Minocycline
NeuN: Neuronal nuclei
PBS: Phosphate buffered saline
PFA: Paraformaldehyde
PI3K: Phosphoinositide 3-kinase
PWT: Paw withdrawal threshold
SMIR: Skin/muscle incision and retraction
TBST: Tris-buffered saline and Tween
TNF: Tumor necrosis factor
WB: Western blot
